# Empirical and Theoretical Modeling of Low-Frequency Noise Behavior of Ultrathin Silicon-on-Insulator MOSFETs Aiming at Low-Voltage and Low-Energy Regime

**DOI:** 10.3390/mi10010005

**Published:** 2018-12-22

**Authors:** Yasuhisa Omura

**Affiliations:** 1 Department of Electrical, Electronics and Information Engineering, Kansai University, Yamate-cho, Suita 564-8680, Japan; omuray@kansai-u.ac.jp; Tel.: +81-663-681-121; 2 Academic Collaboration Associate (ACA), Isehara 259-1135, Japan

**Keywords:** low-frequency noise, silicon-on-insulator, MOSFET, inversion channel, buried channel, subthreshold bias range, low voltage, low energy, theoretical model

## Abstract

This paper theoretically revisits the low-frequency noise behavior of the inversion-channel silicon-on-insulator metal-oxide-semiconductor field-effect transistor (SOI MOSFET) and the buried-channel SOI MOSFET because the quality of both Si/SiO_2_ interfaces (top and bottom) should modulate the low-frequency fluctuation characteristics of both devices. It also addresses the low-frequency noise behavior of sub-100-nm channel SOI MOSFETs. We deepen the discussion of the low-frequency noise behavior in the subthreshold bias range in order to elucidate the device’s potential for future low-voltage and low-power applications. As expected, analyses suggest that the weak inversion channel near the top surface of the SOI MOSFET is strongly influenced by interface traps near the top surface of the SOI layer because the traps are not well shielded by low-density surface inversion carriers in the subthreshold bias range. Unexpectedly, we find that the buried channel is primarily influenced by interface traps near the top surface of the SOI layer, not by traps near the bottom surface of the SOI layer. This is not due to the simplified capacitance coupling effect. These interesting characteristics of current fluctuation spectral intensity are explained well by the theoretical models proposed here.

## 1. Introduction

It has long been considered that there are two possible explanations for the low-frequency noise (LFN) exhibited by metal-oxide-semiconductor field-effect transistors (MOSFETs). They are carrier density fluctuations due to interface traps near the oxide/semiconductor interface [1,2,3], and carrier mobility fluctuations [4,5,6]. Theoretical models have been proposed to comprehensively understand such LFN characteristics (frequently, the 1/f noise) [7,8,9,10,11,12]. Hooge introduced a specific parameter (the so-called Hooge parameter) to characterize the 1/f noise [4]. Related to these theories, the quantum 1/f noise model was proposed by Peter H. Handel [7,8]. However, the physical origins of 1/f noise are not simple and remain controversial [13,14,15] because it is anticipated that the carrier density fluctuation and the carrier mobility fluctuation may be correlated in some cases [11]. In addition, it is considered that the difficulty of understanding the 1/f noise behavior stems from the fact that the Hooge parameter depends on device material and structure [9,15,16].

Although some people have challenged a deeper understanding of the mechanisms of 1/f noise [9,15], clear separation of the aforementioned noise sources, such as carrier density fluctuation and carrier mobility fluctuation, remains rather unclear [15].

The LFN characteristics of various buried-channel MOSFETs (BC-MOSFETs) [17] and various inversion-channel MOSFETs (IC-MOSFETs) [18] have already been discussed in detail based on Hooge’s idea; it was clarified phenomenologically and theoretically that a useful interpretation of the aspects of the Hooge parameter may be possible depending on how the two fluctuation modes (the carrier density fluctuation and the carrier mobility fluctuation) are correlated [17,18]. The gate voltage dependence of the Hooge parameter was explained well by correlating the carrier mobility fluctuation to the carrier density fluctuation. The proposed model gives a valid fundamental physical basis for interpreting various aspects of the Hooge parameter [16,19,20], which suggests that the LFN characteristics of metal-oxide-semiconductor (MOS) devices are not so easily classified

The conventional scaling concept of semiconductor devices has run into the barrier of the cooling limits of very large integrated circuits; the multi-core technology design of integrated circuits and low-power device technology has been proposed because the down-scaling of semiconductor devices is still a goal to permit the greater integration of devices on chips. Such chips must offer low-voltage operation to suppress power dissipation because various sensor networks are created for the purpose of health monitoring and others. Many such sensor devices have to work without any battery. Therefore, high-performance semiconductor devices that can work in a low-voltage condition are needed. Since the low-voltage operation of MOSFETs degrades the signal-to-noise ratio, we have to identify and reduce the various noise sources. In this sense, the physical origin of LFN is now attracting attention because the random telegraph noise (RTN) exhibited by IC-MOSFET memories will be a key determiner of scaled device performance [21,22,23,24,25]. Some articles have theoretically addressed LFN behavior [11,26,27], but very few papers have paid attention to the LFN behavior in the subthreshold bias range [28,29]. In addition, only simplified and qualitative expressions for LFN behavior have been given [30,31,32,33]. Although the conventional model uses just the capacitance coupling effect to express the relation between LFN behavior and both Si/SiO_2_ interfaces [34], we demonstrate here that this simple understanding is incomplete.

In this paper, the LFN behavior of silicon-on-insulator (SOI) IC-MOSFETs and SOI BC-MOSFETs is theoretically revisited from the viewpoint of attaining SOI MOSFETs that can support low-voltage and low-energy applications [35,36]. The SOI layer has two interfaces, and each interface influences the carrier transport and thus LFN. Fortunately, we can investigate the carrier transport of both electrons and holes in the SOI wafer easily by making all SOI layers have the same polarity when MOSFET devices are fabricated on the wafer. In addition, many large scale integration circuits (LSIs) assume this combination from the point of low fabrication cost and design feasibility of threshold voltage operation. Accordingly, this configuration was chosen in this paper. Basically, we concentrate the discussion on the current fluctuation of devices in the subthreshold bias range, and this paper assumes that the LFN behaviors of devices can be characterized by the carrier density fluctuation. This paper also examines on aspects of the drain current fluctuations of short-channel inversion-channel and buried-channel SOI MOSFETs with ultrathin p-Si bodies [37,38,39,40] because it is anticipated that differences in the conduction property will change the noise behavior. First, aspects of the drain current noise behavior are analyzed experimentally in order to categorize the dominant noise sources like interface traps. In addition, physics-based models of the current fluctuation in the subthreshold regime are proposed. Theoretical expressions for LFN behaviors are calculated straightforwardly based on Langevin’s method because we assume the trap-related carrier density fluctuation. The models are validated by measured results and some new findings are discussed. This work will contribute to advances in the device physics of future nano-wire MOSFETs.

## 2. Experiments

**Figure 1:**
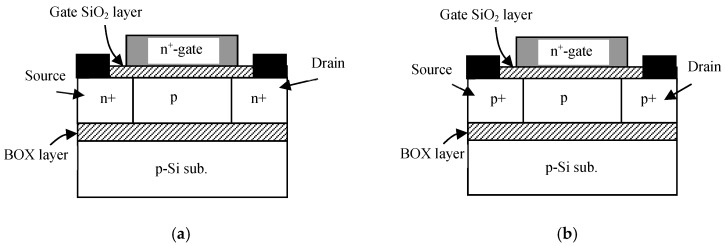
Schematic silicon-on-insulator (SOI) device structures used in experiments. (**a**) n-type inversion-channel (IC) metal-oxide-semiconductor field-effect transistors (MOSFET), (**b**) p-type buried-channel (BC) MOSFET.

A schematic view of the SOI MOS device structures used in the experiments is shown in Figure 1 [41]; the SOI layer (*t_S_*) is 30 nm thick, the gate oxide layer (*t_OX_*) is 7 nm thick, the buried-oxide (BOX) layer (*t_BOX_*) is 80 nm thick, the body-doping concentration (*N_A_*) is 5 × 10^17^ cm^−3^ (n-ch MOSFET) or 4 × 10^17^ cm^−3^ (p-ch MOSFET) of acceptor Boron atoms, and the gate length (*L_G_*) is 0.1 m or 1.0 m. The n-ch MOSFET and p-ch MOSFET have different doping levels (*N_A_*) such that the absolute values of their threshold voltages are nominally the same. The channel length (*L_eff_*) of the 0.1-μm-long gate device is 40 nm, while that of the 1.0-μm-long gate device is 0.95 m. Primary device parameters are summarized in Table 1. The SOI substrates used here were fabricated in the 1990s. Therefore, not only the SOI layer/buried oxide layer interface quality, but also the gate oxide/SOI layer interface quality is only mediocre. As a result, the trap density is larger than expected as is mentioned later.

**Figure 2:**
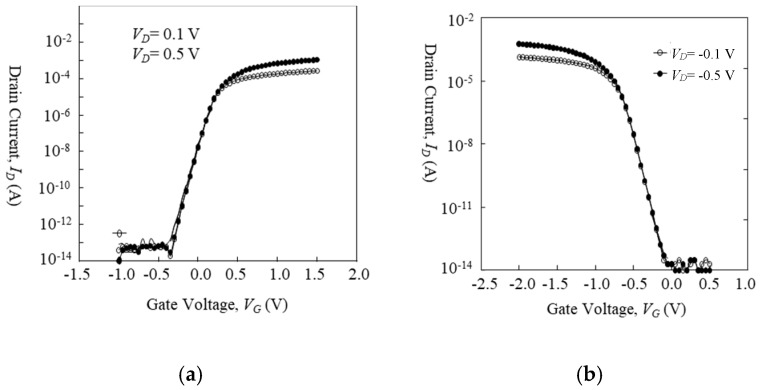
*I_D_* vs. *V_G_* characteristics of 1-μm-long gate MOSFETs. (**a**) n-type IC-MOSFET, (**b**) p-type BC-MOSFET. *V_sub_* = 0 V.

**Figure 3:**
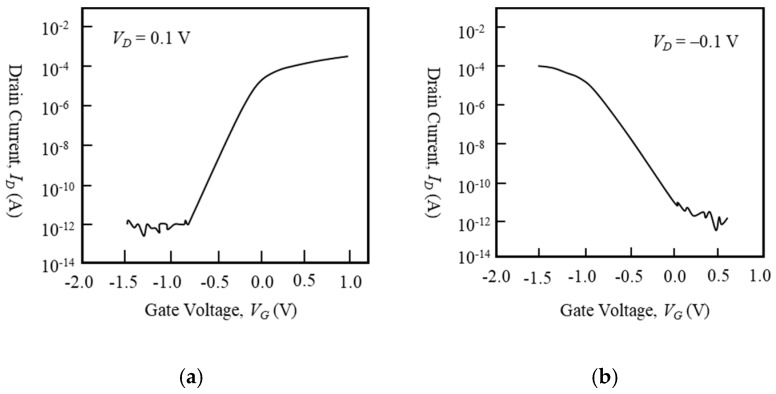
*I_D_* vs. *V_G_* characteristics of 100-nm-long gate MOSFETs. (a) n-type IC-MOSFET, (b) p-type BC-MOSFET. *V_sub_* = 0 V.

Before discussing LFN behaviors, *I_D_* vs. *V_G_* characteristics of devices at the bias condition of LFN measurement are shown in Figure 2 and Figure 3. Figure 2a shows the transfer characteristics of the 1-μm-long gate n-type IC-MOSFET with a 50-μm-long gate width, Figure 2b shows those of the 1-μm-long gate p-type BC-MOSFET with a 50-μm-long gate width, Figure 3a shows that of the 100-nm-long gate n-type IC-MOSFET with a 20-μm-long gate width [41], and Figure 3b shows that of the 100-nm-long gate p-type BC-MOSFET with a 20-μm-long gate width [41]. Although the 100-nm-long gate devices exhibit some slight short-channel effect [41], it does not influence the measurement.

To measure LFN characteristics, the wafer on which the semiconductor devices were fabricated was set on the vacuum chuck. The vacuum chuck was entirely covered with a metal frame to provide electromagnetic shielding; the power supply and current sensing were performed by an Agilent 4156C semiconductor parameter analyzer (Agilent Technologies, Santa Clara, CA, USA) without any preamplifier.

When measuring the drain-current fluctuation of n-channel MOSFETs, +100 mV was applied to the drain terminal. A negative bias was applied to that of the p-channel MOSFETs. The drain current fluctuation was measured from the subthreshold current range to ON-current range. A 900-second measurement of current fluctuation was carried out for every device in order to capture comprehensive sets. Although some people divide the raw data into several parts in order to average them, this paper does not apply the method because it violates the mathematical logic of Fourier transformation. The current level of each device under test was chosen to be higher than 10^−12^ A because the noise current level of the measurement system was ~10^−13^ A in the subthreshold current range.

The following discussions assume that:

(1) $$\frac{S_{I_{D}}\;(f)}{I_{D}{}^{2}}=\frac{C\;(V_{G},\;V_{D}\;,\;V_{sub})}{f^{\gamma }}$$

where *C*(*V_G_*, *V_D_*, *V_sub_*) is a function that depends on the geometrical parameters of devices, gate voltage (*V_G_*), drain voltage (*V_D_*), and substrate voltage (*V_sub_*). Parameter *γ* denotes the exponent of the frequency. In the following section, the measured drain current fluctuations is demonstrated and then theoretical models for the function *C*(*V_G_, V_D_, V_sub_*) are proposed in the subthreshold current range of SOI MOSFETs.

## 3. Results and Discussion

### 3.1. Aspects of the Low-Frequency Noise in Long-Channel Silicon-on-Insulator Metal-Oxide-Semiconductor Field-Effect Transistor (SOI MOSFETs)

**Figure 4:**
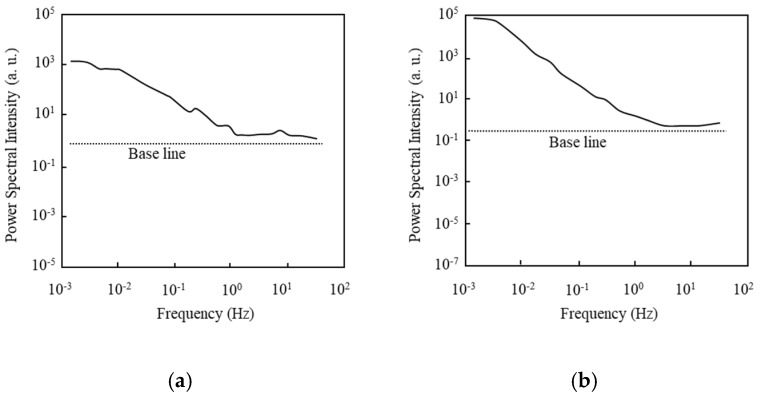
Power spectral intensity of 1.0-μm-long gate SOI MOSFETs at the subthreshold bias. (**a**) nMOSFET (*V_D_* = 0.1 V, *V_G_* = –0.4 V, *V_sub_* = 0 V), (**b**) pMOSFET (*V_D_* = –0.1 V, *V_G_* = –0.4 V, *V_sub_* = 0 V).

First of all, the basic aspects of the drain current fluctuation of the 1-μm-gate SOI MOSFET are investigated. Before characterizing the normalized current fluctuation power spectral intensity ($S_{I_{D}}\;(f)/I_{D}{}^{2}$) of the drain current, the frequency spectra of $S_{I_{D}}\;(f)$ obtained in a subthreshold current range are shown in Figure 4; Figure 4a shows data for the n MOSFET and Figure 4b for the pMOSFET. The baseline is shown to reveal that the value of $S_{I_{D}}\;(f)$ at 0.1 Hz is higher than the background noise level. Each value is different because the measurement condition of the drain current is different. Figure 4 reveals that the value in Equation (1) is larger than unity.

**Figure 5:**
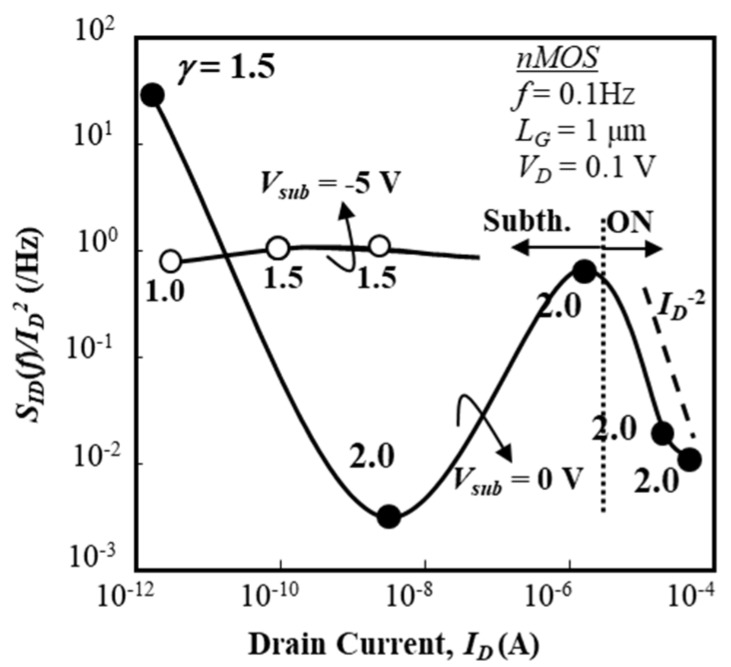
Normalized fluctuation power spectral intensity ($S_{I_{D}}\;(f)/I_{D}{}^{2}$) of drain current as a function of *I_D_* in 1-μm gate IC nMOS. *L_G_* =1 μm and *W_G_* = 50 μm. Substrate voltage (*V_sub_*) is 0 V and –5 V.

Normalized current fluctuation power spectral intensity ($S_{I_{D}}\;(f)/I_{D}{}^{2}$) of the drain current of an n-channel IC-MOSFET as a function of drain current (*I_D_*) is shown in Figure 5 for two substrate bias conditions (*V_sub_* = 0 V, –5 V). In Figure 5, values of $S_{I_{D}}\;(f)/I_{D}{}^{2}$ are extracted from the raw data by the parameter fitting technique and their average values are shown. Since the electric field of SOI/buried-oxide layer interface influences the drain current, numerical simulations were carried out in order to estimate the electric field of the buried-oxide layer. This electric field is estimated, at *V_sub_* = 0 V, to be 2.3 × 10^5^ V/cm at *I_D_* of 10^−12^ A and 2.7 × 10^5^ V/cm at *I_D_* of 10^−6^ A. At *V_sub_* = –5 V, its value is 8.0 × 10^5^ V/cm at *I_D_* of 10^−12^ A and 8.3 × 10^5^ V/cm at *I_D_* of 10^−6^ A. Since these electric field conditions suggest that the SOI/buried-oxide interface does not deplete holes even when *V_sub_* = 0 V due to a small work-function difference between the SOI layer and the substrate, it is expected that electron-related traps around the SOI/buried-oxide interface don’t contribute to the low-frequency noise of the front channel (electron current).

It is seen that $S_{I_{D}}\;(f)/I_{D}{}^{2}$ reveals a deep depression around *I_D_* ~10^−8^ A for *V_sub_* = 0 V. This aspect also appears in sub-micron gate devices (not shown here [29]). On the “ON” state for the gate voltage (*V_G_*) beyond the threshold voltage, $S_{I_{D}}\;(f)/I_{D}{}^{2}$ is proportional to *I_D_*^−*2*^ with *γ* = 2, which strongly suggests that the drain current (electron current) fluctuation is the primary determiner of the carrier density fluctuation due to interface traps [24,25]; it is anticipated that the carrier density fluctuation is due to the interface traps near the top surface of the SOI layer, and that most such traps are effectively shielded by the inversion layer in the “ON” state. On the other hand, $S_{I_{D}}\;(f)/I_{D}{}^{2}$ is basically insensitive to the drain current level (*I_D_*) in the subthreshold bias range for *V_sub_* = –5 V. The behavior of $S_{I_{D}}\;(f)/I_{D}{}^{2}$ for *V_sub_* = –5 V suggests that the interface traps near the bottom surface of SOI layer are effectively shielded by accumulated holes. In contrast, when *V_sub_* = 0 V, the interface traps near the bottom surface of SOI layer are not sufficiently shielded, and some of the electrons contributing to the subthreshold conduction are trapped near the bottom surface. Since the SOI layer thickness is less than the Debye length in this situation, it is expected according to the theoretical model proposed by V. A. Kochelap et al. [42,43] that Coulomb interactions between surface electrons and charged interface traps at the bottom surface may suppress the subthreshold current fluctuation because the surface-noise-suppression factor defined by them increases.

**Figure 6:**
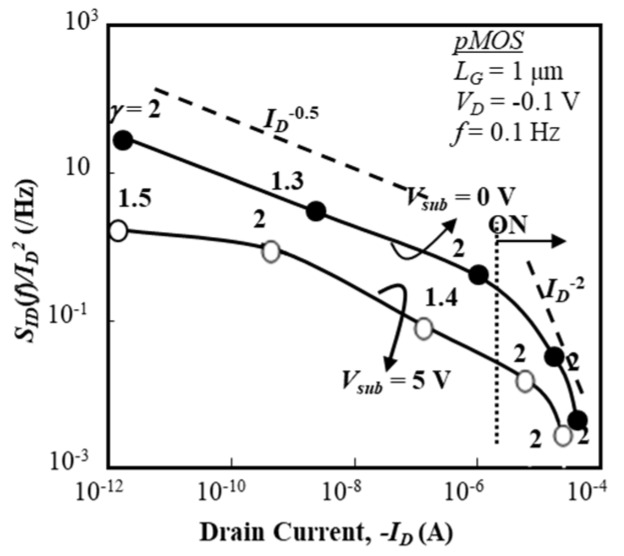
Normalized fluctuation power spectral intensity ($S_{I_{D}}\;(f)/I_{D}{}^{2}$) of drain current as a function of *I_D_* in 1-μm gate BC pMOS. *L_G_* = 1 μm and *W_G_* = 50 μm. Substrate voltage (*V_sub_*) is 0 V and +5 V.

Normalized fluctuation power spectral intensity ($S_{I_{D}}\;(f)/I_{D}{}^{2}$) of the drain current of a p-channel BC-MOSFET as a function of drain current (*I_D_*) is shown in Figure 6 for two substrate bias conditions (*V_sub_* = 0 V, 5 V). In Figure 6, values of ($S_{I_{D}}\;(f)/I_{D}{}^{2}$) are extracted from the raw data by the parameter fitting technique and their average values are shown. Since the electric field of the SOI/buried-oxide layer interface influences the drain current, numerical simulations were carried out for the p-channel BC-MOSFET in order to estimate the electric field of the buried oxide layer. This electric field is estimated, at *V_sub_* = 0 V, to be 1.7 × 10^5^ V/cm at *I_D_* of 10^−12^ A and 3.9 × 10^5^ V/cm at *I_D_* of 10^−6^ A. At *V_sub_* = 5 V, it is 4.0 × 10^5^ V/cm at *I_D_* of 10^−12^ A and 4.4 × 10^5^ V/cm at *I_D_* of 10^−6^ A. When *V_sub_* = 0 V, the effective buried-oxide electric field slightly lowers the threshold voltage of the p-channel BC-MOSFET. When *V_sub_* = 5 V, the electric field of the buried-oxide layer depletes holes from the SOI/buried-oxide interface, which raises the threshold voltage of the p-channel BC-MOSFET; in other words, it is expected that a hole channel will be generated near the top surface of the SOI layer. As a result, it is anticipated that hole-related traps around the SOI/buried-oxide layer do not contribute to the low-frequency noise of the p-channel BC-MOSFET for *V_sub_* = 5 V. Therefore, *V_sub_* dependence of ($S_{I_{D}}\;(f)/I_{D}{}^{2}$) is reasonable.

It is seen that ($S_{I_{D}}\;(f)/I_{D}{}^{2}$) exhibits the drain current dependence of *I_D_*^−0.5^ regardless of substrate bias in the subthreshold bias range, although the magnitude of ($S_{I_{D}}\;(f)/I_{D}{}^{2}$) is reduced if the substrate bias is positive. This suggests that some interface traps of the buried oxide layer do not contribute to the noise because the SOI/buried-oxide layer interface is depleted for *V_sub_* = 5 V. The conventional idea suggests that bulk traps of the SOI layer contribute to the current fluctuation because the buried channel width expands as the gate voltage rises, and/or that interface traps near the top surface of the SOI layer and/or near the bottom surface of the SOI layer contribute to the current fluctuation; however, this is not the case. This behavior, seen in Figure 6, is also observed in sub-micron gate devices (not shown here [29]). In the “ON” state with gate voltages (*V_G_*) beyond the threshold voltage, $S_{I_{D}}\;(f)/I_{D}{}^{2}$ is proportional to *I_D_*^−2^ with *γ* = 2, which strongly suggests that the drain current (hole current) fluctuation is primarily responsible for the carrier density fluctuation due to the interface traps near the top surface of the SOI layer [26,27] because the major part of the hole current consists of the hole accumulation layer near the front gate oxide layer for *V_G_* > *V_TH_*, where *V_TH_* is the threshold voltage. It is considered that the impact of interface traps on the drain current fluctuation is almost the same as that on the inversion channel, although it is anticipated that some traps near the top surface of the SOI layer are shielded by the hole accumulation layer. This consideration is utilized in deriving the theoretical model detailed later.

Past work focused on developing theoretical models for the “ON” state [3,4,5,6,11,12,13,15,16,17,18,19,20,26]. A recent model [27] has been written as:

(2) $$\frac{S_{I_{D}}\;(f)}{I_{D}{}^{2}}={\left(1\pm \frac{\alpha_{c}\mu_{eff}C_{OX}I_{D}}{g_{m}}\right)}^{2}\frac{g_{m}^{2}}{I_{D}^{2}}S_{VFB}(f)$$

(3) $$S_{VFB}(f)=\frac{q^{2}k_{B}T\lambda N_{t}(E_{F})}{W_{eff}L_{eff}C_{OX}^{2}f^{\gamma }}$$

where *α_c_* denotes the scattering factor [11], *λ* denotes the effective tunneling distance, and *N_t_* denotes the effective trap density (cm^−2^∙eV^−1^). Parameter *α_c_* is an empirical parameter, not a physics-based parameter. For the “ON” state shown in Figure 5 and Figure 6, Equations (2) and (3) suggest that trap density *N_t_* is roughly constant in this gate-voltage range for both the IC-MOSFET and BC-MOSFET. This speculation is acceptable because the local Fermi level at the top surface of the SOI layer is still slightly above midgap for the n-ch IC-MOSFET and the local Fermi level at the top surface of the SOI layer is slightly below the Fermi level in the flat-band condition for the p-ch BC-MOSFET. In other words, this suggests that the distribution of *N_t_* over the energy gap of Si definitely controls the behavior of Equation (2).

Although the noise behavior in the subthreshold bias range must be considered, no corresponding physics-based theoretical models have been proposed. This paper corrects this deficiency in a later section.

### 3.2. Aspects of Low-Frequency Noise in 100-nm-long Gate SOI MOSFETs

**Figure 7:**
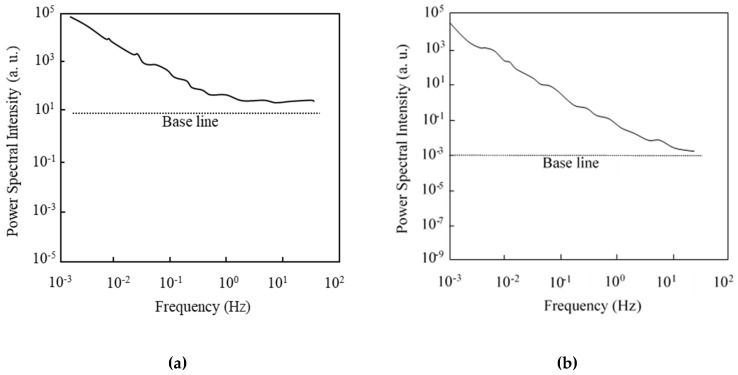
Power spectral intensity of 100-nm-long gate SOI MOSFETs at the subthreshold bias. (**a**) nMOSFET (*V_D_* = 0.1 V, *V_G_* = –0.6 V, *V_sub_* = 0 V), (**b**) pMOSFET (*V_D_* = –0.1 V, *V_G_* = –0.4 V, *V_sub_* = 0 V).

**Figure 8:**
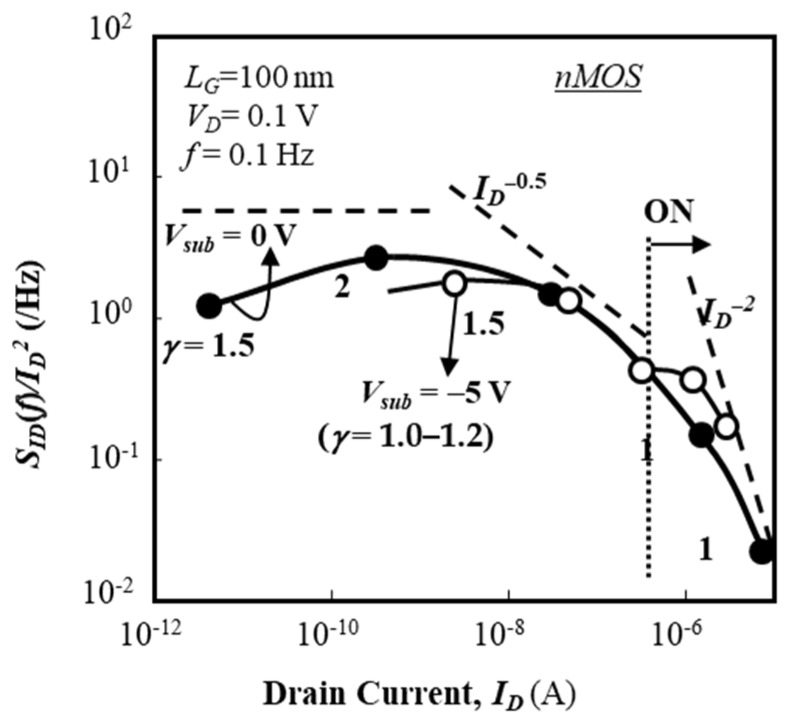
Normalized fluctuation power spectral intensity ($S_{I_{D}}\;(f)/I_{D}{}^{2}$) of drain current as a function of *I_D_* in 100-nm gate IC nMOS. *L_G_* = 100 nm and *W_G_* = 20 μm. Substrate voltage (*V_sub_*) is 0 V and –5 V.

Before characterizing the normalized current fluctuation power spectral intensity ($S_{I_{D}}\;(f)/I_{D}{}^{2}$) of drain current, the frequency spectra of $S_{I_{D}}\;(f)$ obtained in a subthreshold current range are shown in Figure 7; Figure 7a shows the nMOSFET data and Figure 7b shows MOSFET data. Figure 7 reveals that the *γ* value in Equation (1) is larger than unity. Normalized fluctuation power spectral intensity ($S_{I_{D}}\;(f)/I_{D}{}^{2}$) of the drain current of an n-channel IC-MOSFET with 100-nm-long gate (40-nm-long channel) [44] is shown in Figure 8 as a function of drain current (*I_D_*) for two substrate bias conditions (*V_sub_* = 0 V, –5 V). In Figure 8, values of $S_{I_{D}}\;(f)/I_{D}{}^{2}$ are extracted from the raw data by the parameter fitting technique and their average values are shown. $S_{I_{D}}\;(f)/I_{D}{}^{2}$ is insensitive to the drain current in the subthreshold bias range regardless of the substrate bias. The behavior of $S_{I_{D}}\;(f)/I_{D}{}^{2}$ for *V_sub_* = 0 V is very different from that shown in Figure 5; no depression in $S_{I_{D}}\;(f)/I_{D}{}^{2}$ is observed. These behaviors of the 100-nm gate device suggest the following points.

(1) In the subthreshold bias range, the contribution of traps far from the top surface of the SOI layer to the current fluctuation is quite limited, which is anticipated from the insensitivity of $S_{I_{D}}\;(f)/I_{D}{}^{2}$ to the substrate bias. This behavior is different from that of long-channel devices, see Figure 5.

(2) Above the threshold voltage, $S_{I_{D}}\;(f)/I_{D}{}^{2}$ is proportional to *I_D_*^−2^. Equation (2), for example, suggests that the *S_VFB_* factor is roughly constant above the threshold voltage, see Figure 8, when *I_D_* is increased with a constant *V_D_* value because the *I_D_* value is increased when *V_G_* is increased. This suggests that the trap density profile near the midgap is almost flat because the local Fermi level at the top surface of the SOI layer is located around the midgap.

**Figure 9:**
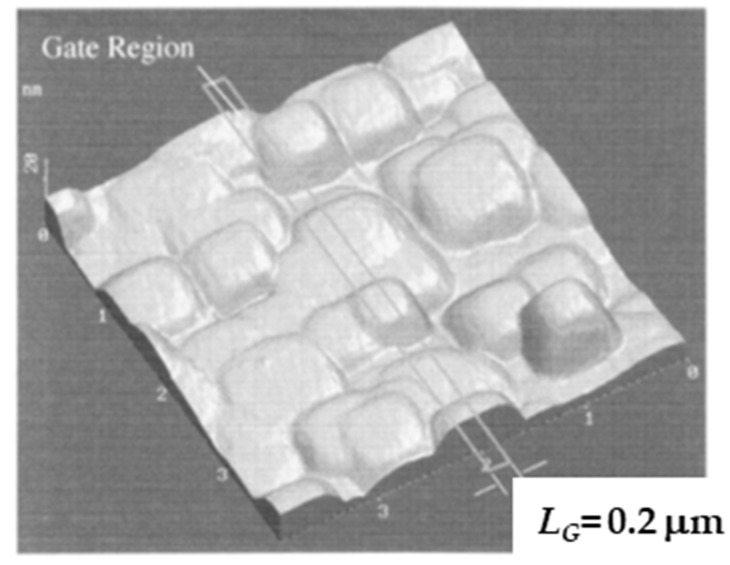
Atomic force microscopy (AFM) image of surface morphology of the bottom surface of the silicon layer. Reproduced with permission from [45], published by IEEE, 1996.

The surface morphology of the buried oxide layer of the MOSFETs used in this experiment has a specific mesa shape aligned to the [100] direction [45,46]; the mesa scale is about 500 nm × 500 nm (in plane) as shown in Figure 9. It is considered that the local fluctuation of the surface potential rules the carrier conduction path in the subthreshold current range [47]. Therefore, it is easily anticipated that the local surface potential of the SOI layer of the MOSFET used here is modified by SOI layer thickness fluctuation [48]. In the present case, it is expected that the spatial uniformity of SOI layer thickness is limited to an area of at most 300 nm × 300 nm, which suggests that the local uniformity of interface trap density is also limited to an area of at most 300 nm × 300 nm. Therefore, the $S_{I_{D}}\;(f)/I_{D}{}^{2}$ behavior of the 100-nm-gate MOSFET is more insensitive to the substrate bias than that of the long-channel MOSFET.

**Figure 10:**
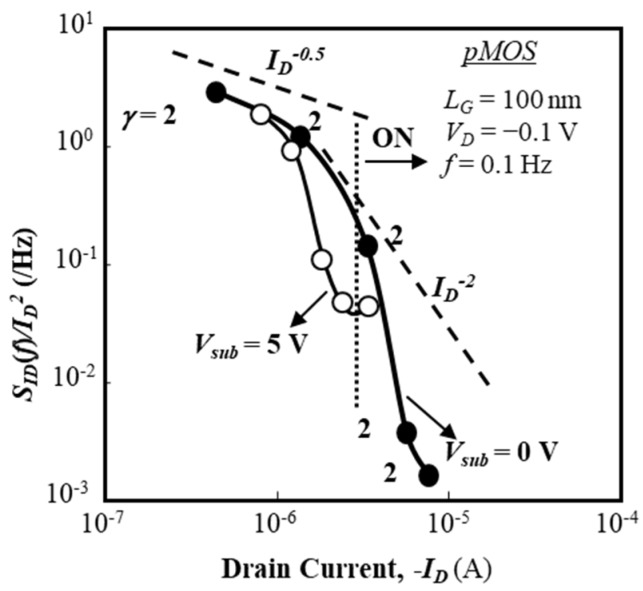
Normalized fluctuation power spectral intensity ($S_{I_{D}}\;(f)/I_{D}{}^{2}$) of drain current as a function of *I_D_* in 100-nm gate BC pMOS. *L_G_* = 100 nm and *W_G_* = 20 μm. Substrate voltage (*V_su_*_b_) is 0 V and +5 V. Reproduced with permission from [49], published by IEEE, 2017.

Next, normalized fluctuation power spectral intensity ($S_{I_{D}}\;(f)/I_{D}{}^{2}$) of the drain current of a p-channel BC-MOSFET as a function of drain current (*I_D_*) is shown in Figure 10 for two substrate bias conditions (*V_sub_* = 0 V, 5 V) [49]. The magnitude of $S_{I_{D}}\;(f)/I_{D}{}^{2}$ shows a weak dependence on *I_D_* (~*I_D_*^−1/2^) in the subthreshold current range regardless of the substrate bias. The behavior of $S_{I_{D}}\;(f)/I_{D}{}^{2}$ is roughly the same as those shown in Figure 6, but it does show a strong dependence on *I_D_* (~*I_D_*^−5^) in the “ON” state. These behaviors of the 100-nm-long gate BC-MOS device suggest the following points.

(1) It is anticipated that the current fluctuation in the subthreshold bias range originates from interface traps near the top surface of the SOI layer, not primarily from interface traps near the bottom interface of the SOI layer.

(2) Following Equation (2), it seems that the trap density energy profile near the top surface of the SOI layer is almost flat, but its value slightly decreases when the local Fermi level of the top surface of the SOI layer approaches the midgap from the level above the midgap because $S_{I_{D}}\;(f)/I_{D}{}^{2}$ decreases (~*I_D_*^−1/2^) as *I_D_* increases.

(3) The contribution of interface traps existing near the bottom surface of the SOI layer to the current fluctuation is not so significant, which is supported by the fact that the magnitude of $S_{I_{D}}\;(f)/I_{D}{}^{2}$ is insensitive to positive substrate bias values. On the other hand, the behavior of $S_{I_{D}}\;(f)/I_{D}{}^{2}$ in the “ON” state reveals that the impact of interface traps near the top surface of the SOI layer on the channel current is greatly suppressed, which is suggested by the very steep decrease in $S_{I_{D}}\;(f)/I_{D}{}^{2}$ in the “ON” state. The channel current formed in the surface hole accumulation layer is basically not influenced by the interface traps near the top surface of the SOI layer because the hole density is very high. This suggests that the surface accumulation layer effectively shields hole traps near the valence band edge.

## 4. Theoretical Modeling for Subthreshold Current Fluctuations

### 4.1. Inversion-Channel MOSFET

Subthreshold current of the n-channel IC-MOSFET at the top surface is given by [50]:

(4) $$I_{D}=I_{Sth}(\phi_{SS})\exp{\left(\frac{q\phi_{SS}}{k_{B}T}\right)}^{2}$$

(5) $$I_{Sth}(\phi_{SS})=\left(\frac{q\mu_{n}W_{eff}V_{D}}{L_{eff}E_{S}(\phi_{SS})}\right)\left(\frac{k_{B}T}{q}\right)\left(\frac{n_{i}^{2}}{N_{A}}\right)$$

where *ϕ*_ss_ denotes the top surface potential, *E_S_* denotes the surface electric field, and we assume *V_D_* <*k_B_T/q*. Other notations follow the conventional terminology. Here, the theoretical formulation follows Langevin’s method [51]. When we assume that the noise source yields the fluctuation of the front surface potential (*ϕ*_ss_), whereas the drain current fluctuation *δI_D_* originates from *δφ_ss_*. Starting with Equation (4), *δI_D_* is given as:

(6) $$\delta I_{D}=\frac{\partial I_{Sth}(\phi_{SS})}{\partial \phi_{{}_{SS}}}\exp\left(\frac{q\phi_{SS}}{k_{B}T}\right)\delta \phi_{SS}+I_{Sth}(\phi_{SS})\exp\left(\frac{q\phi_{SS}}{k_{B}T}\right)\cdot \left(\frac{q}{k_{B}T}\right)\delta \phi_{SS}$$

Then we have,

(7) $$\frac{<\delta I_{D}^{2}>}{I_{D}^{2}}={\left(\frac{q}{k^{B}T}-\frac{1}{2\phi_{SS}}\right)}^{2}<\phi_{SS}^{2}>$$

where <*X*> means the time averaging of the parameter *X*. The spectral density of drain current fluctuation is calculated as:

(8) $$\frac{S_{I_{D}}(f)}{I_{D}^{2}}={\left(\frac{q}{k_{B}T}-\frac{1}{2\phi_{SS}}\right)}^{2}S_{\phi_{SS}}(f)$$

(9) $$S_{\phi_{SS}}(f)=\frac{<{\left(\delta \phi_{SS}\right)}^{2}>}{\Delta f}$$

*S_ϕss_*(*f*) corresponds to the surface potential fluctuation power spectral intensity. It is frequently thought that *S_ϕss_*(*f*) stems from the carrier density fluctuation [2,11]. One possible source of *S_ϕss_*(*f*) is the trapping-detrapping process of carriers near the top surface of the silicon layer. This expression is valid for *ϕ_ss_* > 0; that is *I_D_* >*I_Sth_*.

### 4.2. Buried-Channel MOSFET

On the other hand, the subthreshold current of the p-channel BC-MOSFET near the bottom surface or the top surface is given by [52]

(10) $$I_{D}=I_{Bth}\int_{\phi_{SS}}^{\phi_{BS}}\frac{1}{\sqrt{\phi_{BS}}}\exp\left(-\frac{q\phi_{B}}{k_{B}T}\right)d\phi_{B}$$

(11) $$I_{Bth}=\frac{\mu_{n}W_{eff}V_{D}\sqrt{2\varepsilon_{S}qN_{A}}}{L_{eff}}$$

where *ϕ_SS_* denotes the top surface potential, *ϕ_BS_* denotes the bottom surface potential, and we assume *V_D_* < *k_B_T/q*. Equation (10) can be approximated as:

(12) $$\begin{array}{cc} I_{D} & \approx \frac{1}{2}I_{Bth}(\phi_{SS}-\phi_{BS})\left[-\frac{1}{\sqrt{\phi_{SS}}}\exp\left(-\frac{q\phi_{SS}}{k_{B}T}\right)+\frac{1}{\sqrt{\phi_{BS}}}\exp\left(-\frac{q\phi_{BS}}{k_{B}T}\right)\right]\\ & =\frac{1}{2}I_{Bth}F(\phi_{SS})\left[-\frac{1}{\sqrt{\phi_{SS}}}\exp\left(-\frac{q\phi_{SS}}{k_{B}T}\right)+\frac{1}{\sqrt{\phi_{BS}}}\exp\left(-\frac{q\phi_{BS}}{k_{B}T}\right)\right]\\ & =I_{BS}-I_{SS} \end{array}$$

*F*(*ϕ_SS_*) is given as [41],

(13) $$\begin{array}{ll} \phi_{SS}-\phi_{BS} & =\frac{C_{BOX}}{C_{S}+C_{BOX}}\phi_{SS}-\frac{qN_{A}t_{S}}{2(C_{S}+C_{BOX})}-\frac{C_{BOX}V_{SUB}^{*}}{C_{S}+C_{BOX}}\\ & =F(\phi_{SS}) \end{array}$$

and we have,

(14) $$\frac{d\phi_{BS}}{d\phi_{SS}}=\frac{C_{S}}{C_{S}+C_{BOX}}=1-f_{C}$$

(15) $$f_{C}=\frac{C_{BOX}}{C_{S}+C_{BOX}}$$

Parameter *f_C_* partially represents the capacitance coupling effect. Here *C_S_* denotes the SOI layer capacitance, *C_BOX_* denotes the buried oxide layer capacitance, and V^*^_SUB_ denotes the effective substrate bias.

Assuming that the current fluctuation originates from the traps near the top surface of the silicon, we can say that the surface potential fluctuation directly influences the bottom surface potential fluctuation electrostatically. The theoretical calculation is based on the same approach mentioned in Section 4.1. This argument yields the following expression for the power spectral intensity of the fluctuation of the buried-channel current.

(16) $$S_{ID\_SS}(f)=4{\left(\frac{f_{C}}{F(\phi_{SS})}\right)}^{2}{\left[I_{BS}-I_{SS}\right]}^{2}S_{\phi_{S}}(f)\;+\frac{1}{4}{\left[-I_{SS}\left(\frac{1}{\phi_{SS}}+\frac{2q}{k_{B}T}\right)+2(1-f_{C})I_{BS}\left(\frac{1}{\phi_{BS}}+\frac{2q}{k_{B}T}\right)\right]}^{2}S_{\phi_{S}}(f)\;+f_{C}[I_{BS}-I_{SS}]\left[-\frac{I_{SS}}{F(\phi_{SS})}\left(\frac{1}{\phi_{SS}}+\frac{2q}{k_{B}T}\right)+2(1-f_{C})\frac{I_{BS}}{F(\phi_{SS})}\left(\frac{1}{\phi_{BS}}+\frac{2q}{k_{B}T}\right)\right]S_{\phi_{S}}(f)$$

$S_{\phi_{S}}(f)$ corresponds to the fluctuation power spectral intensity of the top surface potential.

Taking account of the fact that *I_BS_* >> *I_SS_*, Equation (16) can be rewritten as:

(17) $$\frac{S_{ID\_SS}(f)}{I_{D}^{2}}\approx \left\{4{\left(\frac{f_{C}}{F(\phi_{SS})}\right)}^{2}+{\left[(1-f_{C})\left(\frac{1}{\phi_{BS}}+\frac{2q}{k_{B}T}\right)\right]}^{2}\right\}S_{\phi_{S}}(f)+\left\{\frac{2f_{C}(1-f_{C})}{F(\phi_{SS})}{\left(\frac{1}{\phi_{BS}}+\frac{2q}{k_{B}T}\right)}^{2}\right\}S_{\phi_{S}}(f)$$

On the other hand, when it is assumed that the current fluctuation originates from the traps near not only the top surface, but also the bottom surface, of the SOI layer, we have the following expression for the power spectral intensity of the fluctuation of the buried-channel current [51].

(18) $$S_{ID\_SS\_BS}(f)={\left(\frac{I_{D}}{F(\phi_{SS})}\right)}^{2}\left(S_{\phi_{S}}(f)+S_{\phi_{B}}(f)\right)+I_{SS}^{2}{\left(\frac{1}{\phi_{SS}}+\frac{2q}{k_{B}T}\right)}^{2}S_{\phi_{S}}(f)+I_{BS}^{2}{\left(\frac{1}{\phi_{BS}}+\frac{2q}{k_{B}T}\right)}^{2}S_{\phi_{B}}(f)\;-2I_{D}\left(\frac{I_{BS}}{F(\phi_{SS})}\right)\left(\frac{1}{\phi_{BS}}+\frac{2q}{k_{B}T}\right)S_{\phi_{B}}(f)-2I_{D}\left(\frac{I_{SS}}{F(\phi_{SS})}\right)\left(\frac{1}{\phi_{SS}}+\frac{2q}{k_{B}T}\right)S_{\phi_{S}}(f)\;\approx I_{D}^{2}\left(S_{\phi_{S}}(f)+S_{\phi_{B}}(f)\right)+I_{D}^{2}{\left(\frac{1}{\phi_{BS}}+\frac{2q}{k_{B}T}\right)}^{2}S_{\phi_{B}}(f)-2I_{D}^{2}\left(\frac{1}{F(\phi_{SS})}\right){\left(\frac{1}{\phi_{BS}}+\frac{2q}{k_{B}T}\right)}^{2}S_{\phi_{B}}(f)$$

where $S_{\phi_{B}}(f)$ corresponds to the fluctuation power spectral density of bottom surface potential. It is assumed that $S_{\phi_{B}}(f)>>S_{\phi_{S}}(f)$ because *I_BS_* >> *I_SS_*. Thus we have:

(19) $$\frac{S_{ID\_SS\_BS}(f)}{I_{D}^{2}}\approx \left\{\frac{1}{F^{2}(\phi_{SS})}+{\left(\frac{1}{\phi_{BS}}+\frac{2q}{k_{B}T}\right)}^{2}\right\}S_{\phi_{B}}(f)-2\left(\frac{1}{F(\phi_{SS})}\right)\left(\frac{1}{\phi_{BS}}+\frac{2q}{k_{B}T}\right)S_{\phi_{B}}(f)$$

### 4.3. Theoretical Modeling of Fluctuation Sources and Brief Examination of the Model

Following the conventional idea, it can be assumed that the fluctuation source for the current fluctuation originates from the trapping–detrapping process of Si/SiO_2_ interface states [1,2,27]. The top surface potential fluctuation <*ϕ_SS_*^2^> can be written as [2,44]:

(20) $$S_{\phi_{SS}}(f)=\frac{q^{2}k_{B}TN_{t}[E_{F}(\phi_{SS})]\eta^{\gamma -1}}{C_{OX}^{2}W_{eff}L_{eff}f^{\gamma }}$$

where *N_t_*(*E_F_*) denotes the trap density at the local Fermi level, *C_OX_* is the gate oxide capacitance per unit area, and *η* is the parameter (units of frequency) that is used in order to adjust the physical dimension of *S_ϕ__ss_*(*f*). The local Fermi level at the top surface of the SOI layer is a function of the surface potential, *ϕ_SS_*. Other than parameter λ, Equation (20) is basically the same as Equation (3).

In Figure 8, normalized fluctuation power spectral intensity ($S_{I_{D}}\;(f)/I_{D}{}^{2}$) of the drain current of n-channel IC-MOSFETs is almost constant and insensitive to *I_D_* when *V_sub_* = –5 V. Since the bottom surface of the SOI layer is electrostatically shielded by holes when *V_sub_* = –5 V, the electron current near the top surface of the SOI layer is influenced primarily by the interface traps near the top surface of the SOI layer. As Equation (8) is not sensitive to *ϕ_SS_*, Figure 8 suggests that *N_t_*(*E_F_*) is almost flat around the midgap. This speculation is reasonable because *E_F_* approaches the conduction band bottom via the midgap when the gate voltage approaches the threshold voltage.

**Figure 11:**
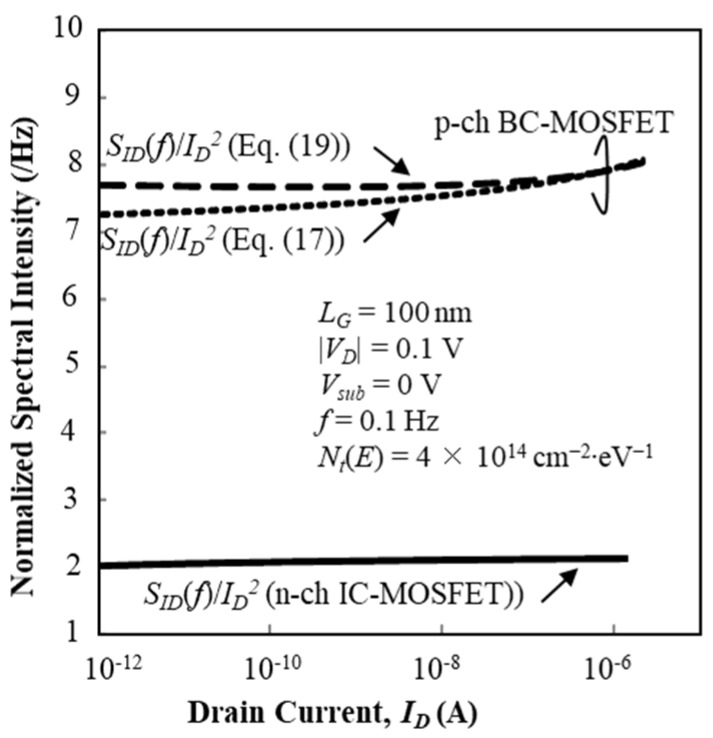
Calculation results of $S_{I_{D}}\;(f)/I_{D}{}^{2}$ as a function of *I_D_* for n-channel IC-MOSFET. It is assumed *N_t_*(*E*) = 4 × 10^14^ cm^−2^∙eV^−1^ and *V_sub_* = 0 V.

**Figure 12:**
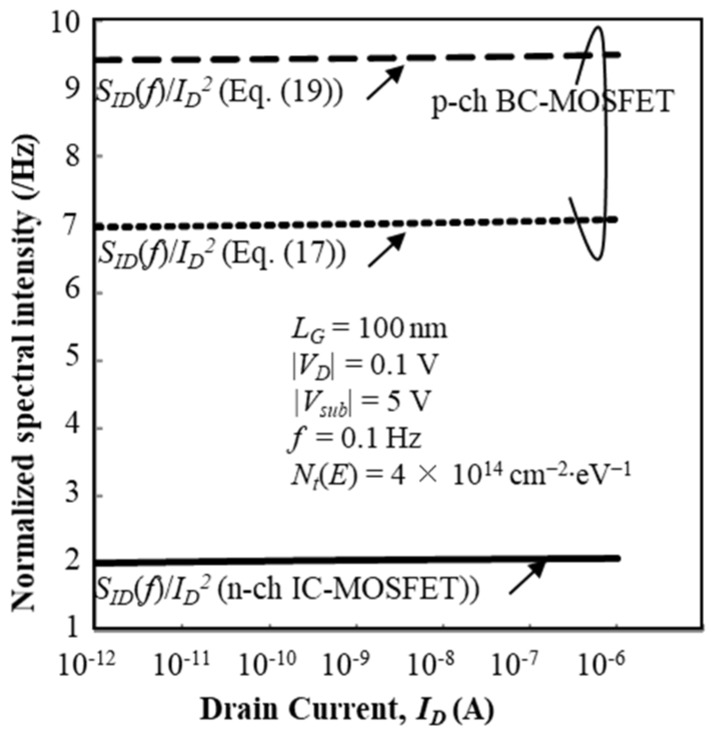
Calculation results of $S_{I_{D}}\;(f)/I_{D}{}^{2}$ as a function of I_D_ for p-channel BC-MOSFET. It is assumed *N_t_*(*E*) = 4 × 10^14^ cm^−2^∙eV^−1^. *V_sub_* = –5 V for nMOSFET and *V_sub_* = +5 V for pMOSFET.

Calculation results of normalized fluctuation power spectral intensity ($S_{I_{D}}\;(f)/I_{D}{}^{2}$) of the drain current of the n-channel IC-MOSFET are plotted as a function of *I_D_* by solid lines in Figure 11 for *V_sub_* = 0 V and Figure 12 for *V_sub_* = –5 V, where it is assumed that *N_t_*(*E*) is constant with the value of 4 × 10^14^ cm^−2^∙eV^−1^. The effective trap density is about 1 × 10^13^ cm^−2^ at room temperature. Calculation results of $S_{I_{D}}\;(f)/I_{D}{}^{2}$ for the n-channel IC-MOSFET are insensitive to *V_sub_*, and the values and behavior insensitivity to *I_D_* well match the experimental results. Therefore, Equation (8) successfully predicts the $S_{I_{D}}\;(f)/I_{D}{}^{2}$ characteristics in the subthreshold bias range.

On the other hand, we show two calculation results of the normalized fluctuation power spectral intensity ($S_{I_{D}}\;(f)/I_{D}{}^{2}$) of the drain current of the p-channel BC-MOSFET as a function of *I_D_* in Figure 11 for *V_sub_* = 0 V and Figure 12 for *V_sub_* = 5 V, where it is assumed that *N_t_*(*E*) is constant with value of 4 × 10^14^ cm^−2^∙eV^−1^. The effective trap density is about 1 × 10^13^ cm^−2^ at room temperature. In Figure 11 and Figure 12, results of Equation (17) are shown by dotted lines and those of Equation (19) are shown by broken lines. $S_{I_{D}}\;(f)/I_{D}{}^{2}$ calculated by Equation (17) decreases with positive substrate bias as seen in Figure 12. However, $S_{I_{D}}\;(f)/I_{D}{}^{2}$ calculated by Equation (19) increases with positive substrate bias, see Figure 12. This assessment reveals that model Equation (17) is acceptable, which means that the current fluctuation of BC-MOSFETs is primarily ruled by the interface traps near the top surface of the SOI layer. This new finding is given by the theoretical analysis proposed in this paper. Equation (17) successfully predicts the $S_{I_{D}}\;(f)/I_{D}{}^{2}$ characteristics in the subthreshold bias range. Finally, we examined whether the value of *N_t_*(*E*) alters the substrate bias dependence of $S_{I_{D}}\;(f)/I_{D}{}^{2}$ assuming the adoption of Equations (17) and (19). When *N_t_*(*E*) = 4 × 10^13^ cm^−2^∙eV^−1^, $S_{I_{D}}\;(f)/I_{D}{}^{2}$ values are reduced to one tenth that for *N_t_*(*E*) = 4 × 10^14^ cm^−2^∙eV^−1^ because the surface potential fluctuation is proportional to *N_t_*(*E*) (see Equation (20)). However, the substrate bias dependence of $S_{I_{D}}\;(f)/I_{D}{}^{2}$ does not change.

## 5. Conclusions

This paper elucidated the normalized drain current fluctuation spectral intensity of various long-channel and short-channel SOI MOSFETs (inversion-channel SOI MOSFETs and buried-channel SOI MOSFETs) from the viewpoint of scaling. This paper reconsidered low-frequency noise behavior in the inversion-channel SOI MOSFET and the buried-channel SOI MOSFET because it is anticipated that the quality of both Si/SiO_2_ interfaces should modulate the low-frequency noise characteristics of both devices. Our assessments also addressed the low-frequency noise behavior of sub-100-nm-long channel SOI MOSFETs. The low-frequency noise behavior in the subthreshold bias range was discussed in some detail in order to consider device suitability for future low-voltage and low-power applications.

This paper also proposed theoretical models to explain and predict the drain current fluctuations of SOI MOSFETs. For the buried-channel device, two models were proposed; one assumes that the drain current fluctuation originates from the traps near the top surface of the SOI layer, and the other assumed that the drain current fluctuation originates from the traps near the bottom surface of the SOI layer. As expected, the analyses showed that the current fluctuation of the inversion channel SOI MOSFET is strongly influenced by interface traps near the top surface of the SOI layer because those traps are not well shielded by surface weak inversion carriers in the subthreshold bias range. However, unexpectedly, the buried channel is primarily influenced by interface traps near the top surface of the SOI layer, not by traps near the bottom surface of the SOI layer. This interesting characteristic of current fluctuation spectral intensity was well explained by the theoretical models proposed here. One theoretical expression reveals that the impact of substrate bias is not due to just the capacitance coupling effect, which contradicts the conventional model. As a result, the interface trap density of the top surface of the SOI layer should be reduced in order to improve the analog performance of SOI MOSFETs.

## Figures and Tables

**Table 1:** Parameters of fabricated devices.

Devices	*t_s_*	*t_ox_*	*t_BOX_*	*N_A_*
IC-MOSFET	30 nm	7 nm	80 nm	5 × 10^17^ cm^−3^
BC-MOSFET	30 nm	7 nm	80 nm	4 × 10^17^ cm^−3^
